# Scaling Up Molecular Electron Spin Qubits Around a Core‐Shell Quantum Dot

**DOI:** 10.1002/chem.71619

**Published:** 2026-08-29

**Authors:** Simon G. McAdams, Jesus Ferrando‐Soria, Paul D. McNaughter, Edward A. Lewis, Sarah J. Haigh, David J. Lewis, Richard E. P. Winpenny, Paul O'Brien, Floriana Tuna

**Affiliations:** ^1^ Department of Chemistry University of Manchester Manchester UK; ^2^ Department of Materials University of Manchester Manchester UK; ^3^ National Graphene Institute University of Manchester Manchester UK; ^4^ Photon Science Institute University of Manchester Manchester UK

**Keywords:** EPR, molecular nanomagnets, molecular qubits, quantum dots

## Abstract

Attachment of molecular nanomagnets (MNMs) to surfaces is an essential step toward multi‐qubit assemblies for the implementation of quantum information technologies. Here, thiol‐monofunctionalized {Cr_7_Ni} molecular antiferromagnetic rings that have potential as electron spin qubits have been synthesized and successfully attached to the surface of a highly luminescent core‐shell CdSe/ZnS quantum dot (QD) nanoparticle via a ligand exchange process. The successful grafting of eleven molecular rings per QD was confirmed by high‐angle annular dark field (HAADF) scanning transmission electron microscope (STEM) imaging, energy dispersive x‐ray (EDX) imaging, and dynamic light scattering (DLS) measurements. Photoluminescence (PL) and pulse electron paramagnetic resonance (EPR) spectroscopies indicated that the resulting hybrid nano‐objects display the bright optical emission provided by CdSe/ZnS QDs and long‐lived quantum coherence associated with eleven grafted {Cr_7_Ni} molecular qubits. Thus, grafting of MNMs to nanoparticle surface provides an elegant method for generating hybrid assemblies with magnetic optical dual functionality and a facile approach to scale electron spin qubits.

## Graphical abstract

Making assemblies of molecular nanomagnet (MNM) qubits is an essential step toward scalable quantum devices. Here, SH‐functionalized {Cr_7_Ni} antiferromagnetic rings with ability as MNM qubits were successfully grafted to the surface of fluorescent CdSe/ZnS quantum dots (QD). The resulting hybrid is intensely fluorescent and magnetic showing robust quantum coherence associated with eleven chemically grafted {Cr_7_Ni} qubits.

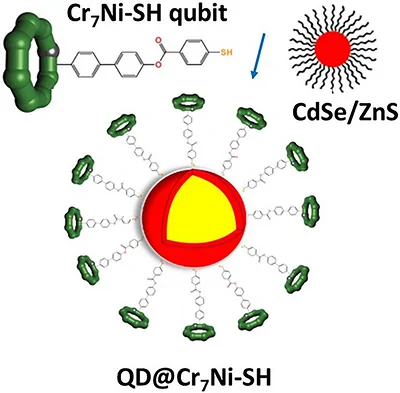

## 1 Introduction

Quantum computing (QC) has the potential to significantly enhance computation rates by utilizing quantum mechanics to process computation in parallel rather than sequentially. The central concept is that information is encoded in a two‐state quantum register known as a *qubit* (or quantum bit), which has the ability to utilise and control a large number of superposition states that are additional to the common 0 and 1 used in classical computation (CC) [1]. With such an increased power, QC is expected to solve complex algorithms and answer questions that are currently unachievable with the clasical computing [2]. Many possible qubits have been proposed, including quantum dots (QD) [3, 4], carbon nanotubes [5], trapped ions [6], nuclear spins [7], molecular spins [8, 9, 10], and molecular nanomagnets (MNMs) [11, 12, 13, 14, 15]. Electron spin‐bearing molecules are particularly promising as potential qubits due to their high tunability and scalability [2, 16], coupled with ability to be manipulated by electromagnetic radiation using electron paramagnetic resonance (EPR) [17, 18]. Indeed, several EPR‐active metal complexes and organic radicals demonstrated long‐lived quantum coherence that classifies them as potential qubits [8, 19, 20, 21, 22, 23, 24, 25]. Moreover, significant progress has been made toward the design of optically addressable molecular qubits [26, 27]. However, the research has been essentially focused on compounds that display a ground‐state spin that could be initialised and read out using light, and coherently manipulated using microwaves. Despite such progress, there are important chalenges to still be addresed. For instance, execution of computation alghoritms requires the formation of multi‐qubit gates fulfilling the so‐called DiVincenzio criteria [28], and of spin‐optical or spin‐electrical interfaces to enable more efficient iniatialisation and read‐out. While studies of two‐qubit molecules now exist, with proposals to perform the CNOT [29, 30, 31, 32, 33, 34, 35] and √iSWAP gates [36, 37], there are fewer reports of larger molecules involving multiple spin centers interacting weakly and/or coherently [32, 38], though the implementation of adiabatic QC with three electronic qubits hosted in one supramolecule was demonstrated experimentally using EPR [39].

Of special interest are MNMs based on octametallic molecular rings such as [^n^Pr_2_NH_2_][Cr_7_Ni(μ‐F)_8_(O_2_CtBu)_16_], abbreviated as {Cr_7_Ni} (**1**) (Figure 1), which possess a *S* = ½ molecular ground state due to strong intramolecular antiferromagnetic coupling between seven Cr^3+^ (3d^3^, *s* = 3/2) and one Ni^2+^ (3d^8^, *s* = 1) ions [40], with long enough phase memory times to be considered viable qubit candidates [11]. The qubit lifetime of the respective molecular rings can be further optimized by ring functionalisation, solvent deuteration and/or magnetic dilution, unlocking remarkable qubit memory times (>15 µs at 1.5 K [41]). Moreover, the high stability of these {Cr_7_Ni} rings makes it possible to functionalise them in a controlled way to create multi‐ring supramolecular arrays of desired architecture. In recent previous work, {Cr_7_Ni} rings were used as building blocks to form large assemblies of rings [42, 43, 44] and molecular quantum gate prototypes that could execute quantum logic operations [30, 45, 46].

**FIGURE 1:**
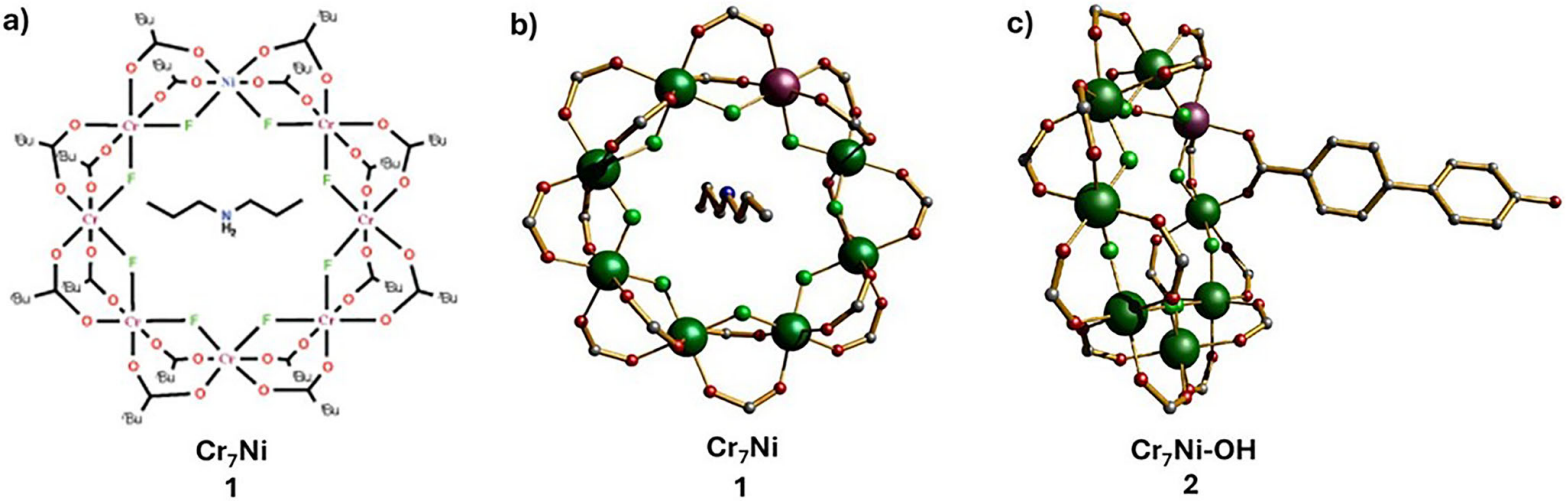
(a) Molecular structure of {Cr_7_Ni} ring (**1**), with Pr_2_NH_2_ as the templating cation; (b) Crystal structure of the molecule 1 presented in (a), with H atoms and tertiary butyl groups from the carboxylate bridging ligand omitted for clarity; and (c) Crystal structure of **2**, with Pr_2_NH_2_ templated cation, H atoms and tertiary butyl groups omitted for clarity. Color code: Cr, green; Ni, purple; N, blue; O, red; C, grey; F, pale green.

The possibility of forming multi‐qubit assemblies that ensure weak interactions between qubit components is encouraging as it opens opportunities to transition toward scalable quantum devices. We recall here that a weak and controlled inter‐qubit interaction is a key prerequisite of quantum devices to ensure entanglement while minimizing unwanted cross‐communications and decoherence [28]. We have previously approached this challenge in two ways: first, by using supramolecular capsules developed by Fujita and colleagues, and building an array of twenty‐four {Cr_7_Ni} qubits around this capsule [43]. Second, we have built oligomeric rotaxanes where through the functionalization of the organic cation backbone have reached an assembly containing six qubits [44].

Here we have explored the possibility of attaching {Cr_7_Ni} MNMs to a preformed nanoparticle with the scope of forming multi‐qubit assemblies supported on an optical and semiconducting platform. We hypothesize that a nanoparticle such as a QD, which is both semiconducting and strongly luminescent [47], is the ideal platform for scaling up MNM qubits, offering the prospect in the long term to utilize its (photo)physics for addressing the spins of the MNMs. By engineering Cr_7_Ni@QD hybrids one can exploit the advantages of both molecular science and quantum photonics for the benefit of quantum information processing (QIP), considering that QDs are themselves potential qubits, with examples spanning from Mn^2+^‐doped ZnO, ZnSe, CdSe, and PbSe QDs [48, 49, 50] to Si/SiGe nanostructures [51]. Their photoluminescent emission can be controlled by adjusting their size during synthesis, with the smaller dots emitting blue‐shifted light, while the larger dots emitting red‐shifted light [52]. Recently, some of us and others demonstrated the possibility of single Mn^2+^ ion insertion into a core‐shell QD nanocrystal, InP/ZnSeS [4] or InGaAs/GaAs [53], with the new nanoparticle demonstrating prolonged spin‐lattice and spin‐coherence lifetimes along with high luminescence quantum yield (QY). Other studies have shown that nanoparticles doped instead with rare earth ions show photo‐switching behavior that can be promising for QIP [54]. Colloidal QD nanoparticles are also appealing as single‐photon emitters [55], individual nanocrystal semiconductors [56], and substrate‐free, nano‐positionable platforms offering possibilities to scale qubits to millions if necessary for QIP applications [57].

Bulk surfaces have been widely investigated as substrates for MNMs [58, 59, 60, 61]. For example, we have previously grafted {Cr_7_Ni} molecular antiferromagnetic rings to an Au(111) substrate using thiol functional groups [60, 61]. However, few studies exist on the attachment of MNMs to nanoparticles. This is surprising considering that nanoparticles have a large surface area that could allow a high loading of MNMs and formation of large assemblies and multimodal hybrids. Previous work has attached either Fe_4_ or Dy_2_ MNMs to gold nanoparticle surfaces, finding in both cases a decrease in the MNM spin reversal energy barrier [62, 63]. Akin to gold nanoparticles, semiconductor nanocrystals known as “quantum dots” (QDs) are ideal scaffolds for MNMs, owing to an amenable surface that can accommodate a broad variety of capping ligands [64], in addition to having a bright and size‐tuneable optical emission [47].

In the present study, a novel {Cr_7_Ni} derivative possessing a single pendant thiol group, abbreviated as {Cr_7_Ni‐SH} (**3**) was successfully grafted to the surface of an octadecylamine capped core‐shell CdSe/ZnS QD as an efficient route to generate multi‐qubit bimodal nanocrystals (Figure 2). The resulted nano‐objects display robust quantum coherence and intense photoluminescence (PL). It is worthy to note that a controllable chemical design of a monofunctionalized {Cr_7_Ni} with a pendant arm is of key importance to avoid agglomeration of the QDs post ligand exchange. Previous ligand exchanges with bifunctional Fe_4_ and TbPc_2_ MNMs onto gold nanoparticles resulted in uncontrolled agglomeration [59, 60]. Equally important is the coating of the CdSe core with a ZnS passivation layer that enhances the photo‐stability of the QD and prevents the degradation of its core. This was demonstrated by us in a previous study where we achieved unprecedented relaxivity and enhanced PL QYs through the grafting of thiol‐functionalized Gd‐based MRI contrast molecules onto the surface of ZnS‐passivated core‐multishell CdSe/CdS/ZnS QDs [65]. Indeed, we observe a red‐shift and nearly threefold increase in PL QY after shelling CdSe with two ZnS monolayers. The successful grafting of {Cr_7_Ni} rings is probed unambiguously by a suite of powerful complementary techniques including high angle annular dark field (HAADF) scanning transmission electron microscope (STEM) imaging, energy dispersive x‐ray (EDX) imaging, and dynamic light scattering (DLS) measurements. Most importantly, these rings preserve their qubit properties, which is of outmost importance in view of quantum information processing applications.

**FIGURE 2:**
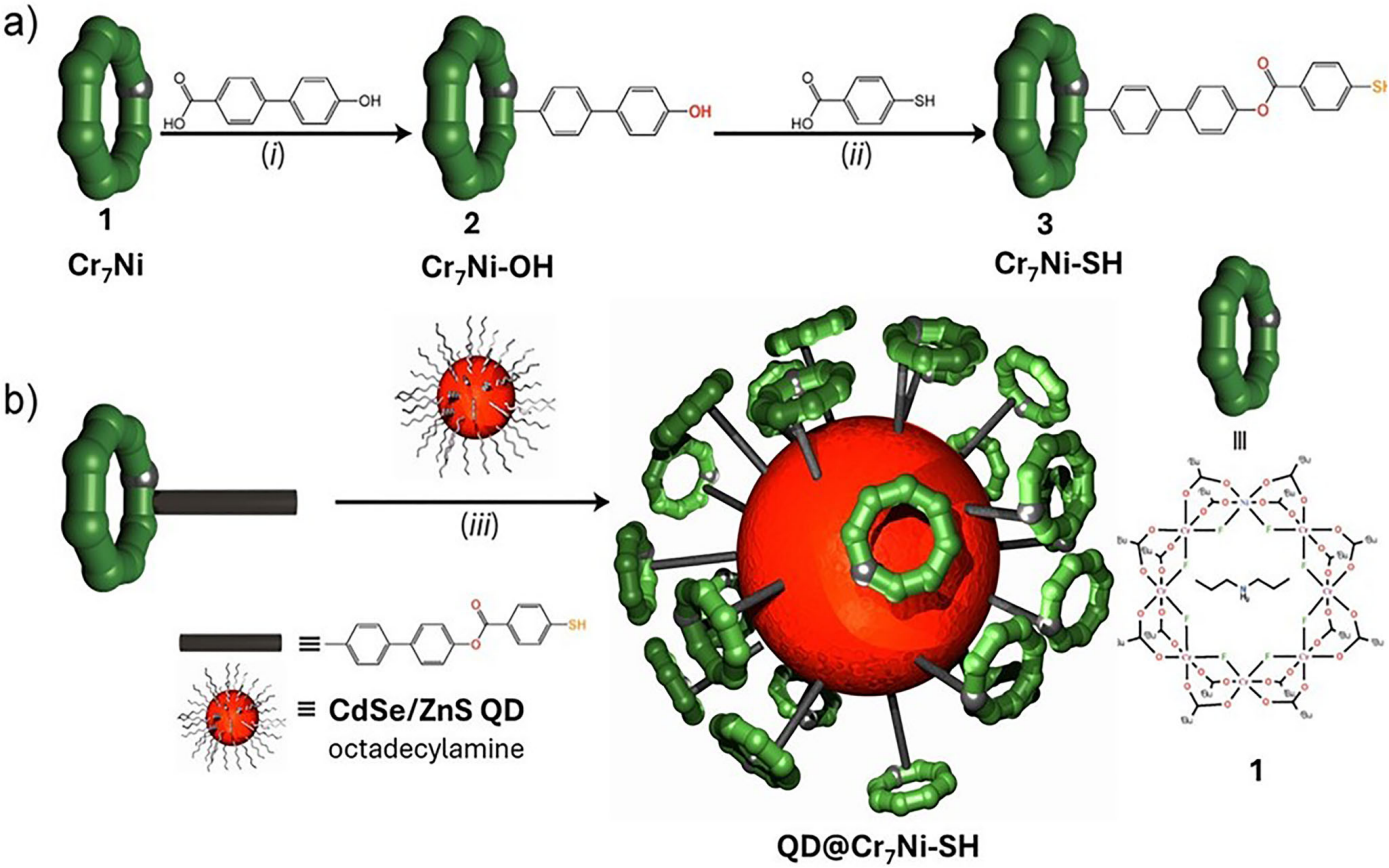
Schematic of the thiol functionalization of {Cr_7_Ni} rings and subsequent formation of QD@Cr_7_Ni‐SH: (a) Synthesis of key component **3**: (i) toluene, 1,2‐dicholorobenzene, and dimethyformamide with stirring at 150°C for 12 hrs; (ii) dicyclohexylcarbodiimide, 4‐dimethylaminopyridine in THF with stirring at room temperature for 48 hrs under Ar gas. (b) Grafting of **3** to the surface of an octadecylamine coated CdSe/ZnS QD via the thiolate group: (iii) chloroform, with stirring at room temperature for 24 hrs under N_2_ gas, then acetone followed by centrifugation.

## 2 Results and Discussion

### 2.1 Synthesis

In this work we explored the possibility of anchoring {Cr_7_Ni} heterometallic rings to the surface of a nano‐size QD via an organic linker that keeps the ring at a sufficient distance to prevent strong magnetic interactions between rings, which is a necessary step toward QC implementation. We hypothesized that a functionalized {Cr_7_Ni} ring with a single pedant thiol group, abbreviated as {Cr_7_Ni‐SH} (**3**) will allow to achieve this goal (Figure 2a). To synthesize {Cr_7_Ni‐SH}, we started from a chemically engineered heterometallic octanuclear coordination cage, [*^n^* Pr_2_NH_2_][Cr_7_Ni(μ‐F)_8_(O_2_C^t^Bu)_16_] (**1**), hereafter abbreviated as {Cr_7_Ni} [40]. A key design feature is the very high solubility of **1** and its derivatives in noncoordinating solvents. This feature, which is easily overlooked, allows the production of very large molecules without any interference from coordinating or protic solvent and without complications caused by insolubility of side products [43]. We first performed a selective carboxylate substitution reaction on **1** [42, 46], which has allowed to introduce 4’‐hydroxy‐4‐biphenylcarboxylic acid on a Cr···Ni edge, giving {Cr_7_Ni‐OH} (**2**) in high yield [43] (Figures 1c and 2a).

In a subsequent step, compound **2** was reacted with 4‐mercaptobenzoic acid via a Steglich esterification reaction between the free alcohol and carboxylic acid to yield {Cr_7_Ni‐SH} (**3**) in 55% yield (see Experimental Section). The 3.4 nm CdSe QD cores were synthesized [66] and shelled with two monolayers of ZnS using the SILAR method and a previously reported protocol [67]. Core‐shell CdSe/ZnS QD nanocrystals coated with octadecylamine were isolated by centrifugation, repeatedly washed with chloroform and kept at room temperature under N_2_. As expected, the size of the nanoparticles has increased, supporting the successful addition of two monolayers of ZnS. Additionally, the newly synthesized core‐shell CdSe/ZnS nanoparticles demonstrated a red‐shift in PL by 8 nm, compared to the unshelled CdSe cores, and became significantly brighter, experiencing a notable increase in PL QY from 5.9 % in the 3.4 nm CdSe cores to 15.1% in CdSe/ZnS QDs. Thus, the passivation effect of ZnS positively impacted the optical properties of the nanocrystals.

For the ligand exchange, an excess of {Cr_7_Ni‐SH} (**3**) was stirred with CdSe/ZnS QDs in chloroform for 24 h under N_2_ gas. An excess of acetone was added to precipitate the QDs functionalized with molecular {Cr_7_Ni‐SH} rings, denoted forthwith as QD@Cr_7_Ni‐SH. These hybrid nanocrystals were isolated via centrifugation, washed repeatedly with acetone and finally suspended in CHCl_3_ for further characterization.

### 2.2 Transmission Electron Microscopy

To confirm the attachment of {Cr_7_Ni‐SH} to the surface of CdSe/ZnS QDs, QD@Cr_7_Ni‐SH particles were imaged using high angle annular dark field (HAADF) STEM imaging with energy‐dispersive X‐ray (EDX) spectrum imaging (Figure 3). The particles appeared pseudo‐spherical (Figure 3a,c) with a wurtzite crystal structure (Figure 3b), and were 4.2 ± 0.3 nm in diameter (Figure 4d). EDX spectra confirm the presence of the constitute elements of the CdSe/ZnS core‐shell QDs (Figure 3g), including the presence of Cr, attributed to the {Cr_7_Ni‐SH} rings. As expected, a Cr signal is absent in comparable unfunctionalized QDs (Figure S1b). Elemental maps extracted from the spectrum images confirm the QD's core‐shell structure (Figure 3e) and show that Cr is indeed peppered over the surface of the CdSe/ZnS QDs (Figure 3f,h). The individual elemental maps for other constituent elements are shown in Figures 3h and Supplementary Information Figure S1.

**FIGURE 3:**
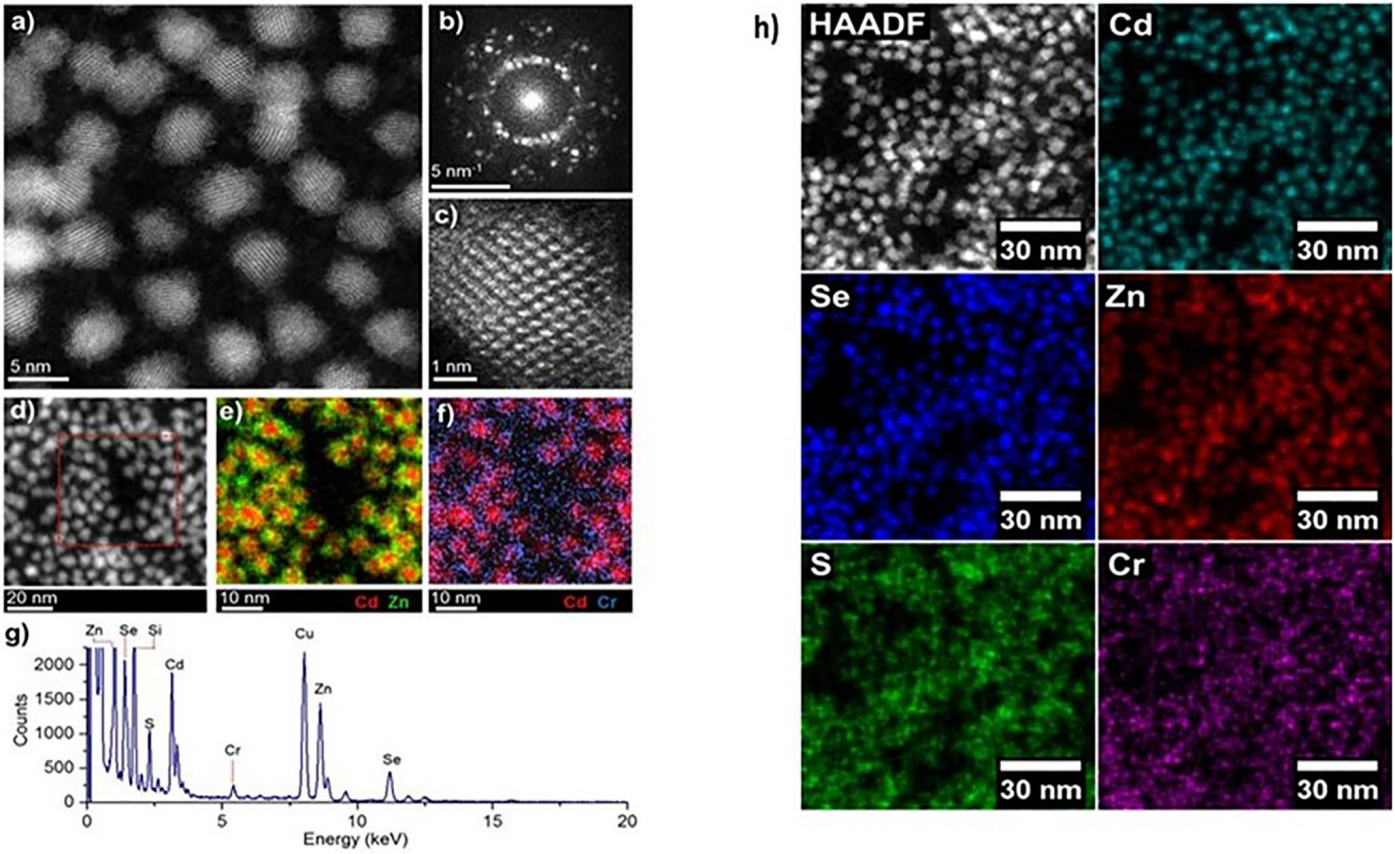
(a) HAADF STEM image of CdSe/ZnS@Cr_7_Ni‐SH QD at atomic resolution; (b) Fourier transform (FT) of (a), all spots can be indexed to Wurtzite CdSe; (c) magnified image of a single QD from (a); (d) low‐magnification HAADF STEM image with the region studied by EDX spectrum imaging indicated by dashed red line; (e) and (f) show elemental maps extracted from the spectrum image showing distributions of Cd (red) and Zn (green), and Cd (red) and Cr (blue) respectively; (g) summed EDX spectrum extracted from the spectrum image, and (h) HAADF‐STEM image of QD@Cr7Ni‐SH (top left) and corresponding EDX elemental maps for Cd, Se, Zn, S, and Cr.

**FIGURE 4:**
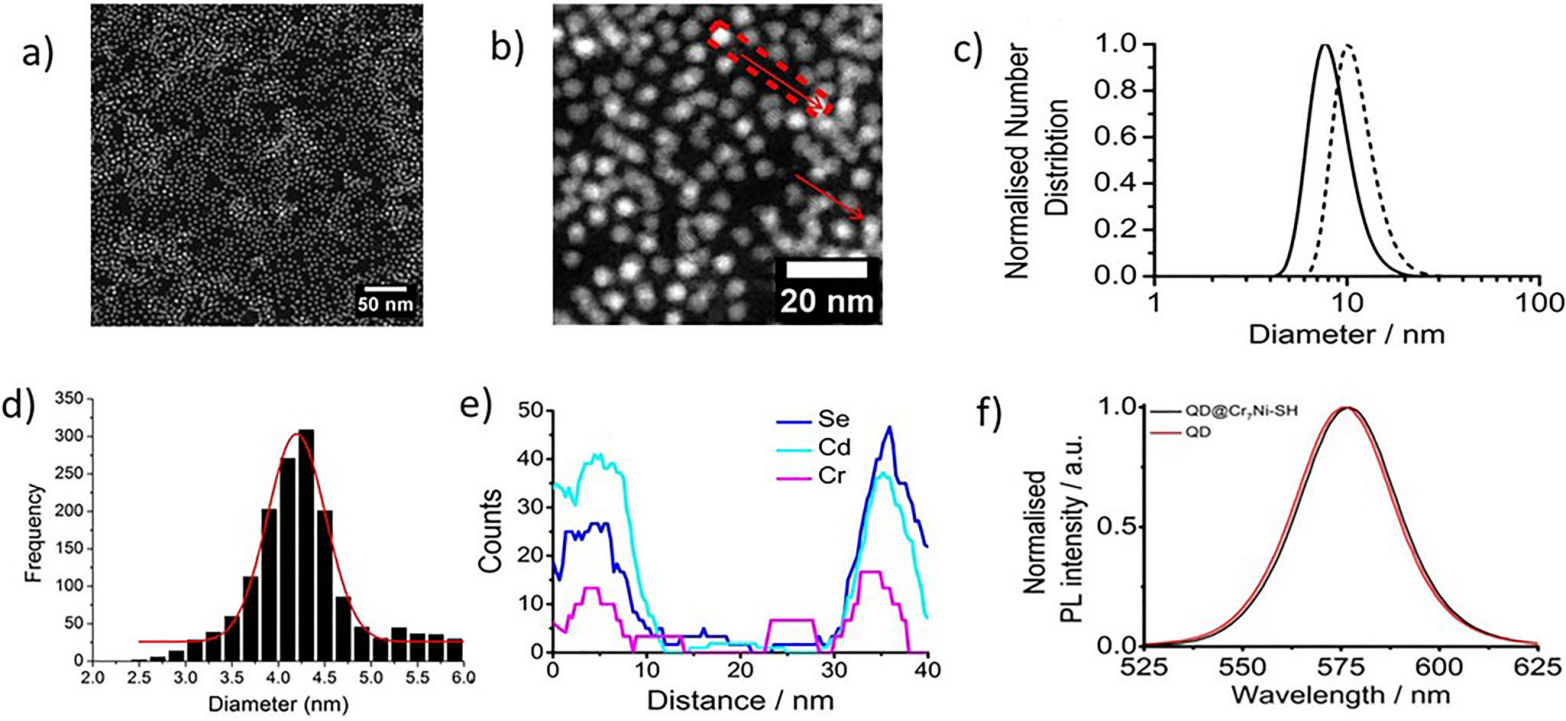
(a) HAADF STEM image of QD@Cr_7_Ni‐SH, with (d) corresponding histogram of particle size distribution with Gaussian fit (red line). Mean particle size was measured as 4.2 ± 0.3 nm. (b) a magnified area of HAADF STEM image of QD@Cr_7_Ni‐SH with marked area that was indexed by EDX, arrows marking the scan direction, and (e) the corresponding EDX line‐profile spectrum of Cd (red); Se (black) and Cr (green). (c) Number percentage distribution of QDs and QD@Cr_7_Ni‐SH in chloroform obtained through DLS before (black) and after {Cr_7_Ni‐SH} ligand exchange (dashed). (f) Normalized PL spectra (λ_exc_ = 400 nm) of QDs (red) and QD@Cr_7_Ni‐SH (black) in chloroform. Dotted lines represent maxima of the respective peaks; and the arrow the direction of the PL shift. All spectra are corrected for instrument response.

To further probe Cr_7_Ni attachment to the QD, DLS measurements were performed, showing an increase in the hydrodynamic diameter from 8.5 to 11.3 nm before and after {Cr_7_Ni‐SH} ligand exchange, respectively (Figure 4c). This increase in hydrodynamic diameter of 2.8 nm is consistent with {Cr_7_Ni‐SH} attachment. Using inductively coupled plasma optical emission spectrometry, a ratio of 1 QD to *ca*. 11 {Cr_7_Ni‐SH} rings was calculated. This is a reasonable estimate considering that approximately 15 {Cr_7_Ni‐SH} rings can bind to one QD based on the ligand's ‘footprint’ (Section S5 for calculation details), and that not all binding sites will be available due to the presence of some irreversibly bound hexadecylamine molecules [68].

### 2.3 Photoluminescence Spectroscopy

PL spectroscopy has revealed a 1 nm red‐shift in the emission wavelength of QD@Cr_7_Ni‐SH compared to the as synthesized CdSe/ZnS QDs (λ_exc_ = 400 nm) (Figure 4f). Considering that the full width at half maximum (FWHM) is 29 nm for both materials, the red‐shift is indicative of the interaction between the {Cr_7_Ni‐SH} rings and the QD surface. A similar PL red‐shift has been reported as a result of a ligand exchange with thiol‐containing capping ligands, possibly due to increased delocalization of the electron‐hole wavefunction [69]. The fluorescence QY was measured as 15.1 ± 1 % and 16.2 ± 1% for QDs and QD@Cr_7_Ni‐SH, respectively. The fact that the hybrid material remains luminescent is interesting, considering that thiol interactions with CdSe QDs typically cause fluorescence quenching [67, 70, 71, 72]. However, such quenching is often observed in aqueous colloidal solutions where surface thiolates (R‐S·) act as hole traps [73]. As our materials are dispersed in chloroform, this effect is likely minimized. Moreover, it has been demonstrated that thiol addition in low concentration can improve passivation of electron traps, yielding in return an increased QY [73, 74]. Considering that the only thiol groups binding to CdSe/ZnS QDs are those of {Cr_7_Ni‐SH} rings that are grafted onto the QD surface, it is reasonable to assume that the observed enhancement in luminescence QY for QD@Cr_7_Ni‐SH is due to a surface passivation effect.

### 2.4 EPR Spectroscopy

With the successful grafting of Cr_7_Ni‐SH to the CdSe/ZnS QD surface, it was necessary to probe that the new hybrid retained the magnetic and spin relaxation properties associated with the grafted Cr_7_Ni‐SH MNMs. Specifically, we wished to confirm that the characteristic *S* = ½ ground state and the long lived quantum coherence properties of the molecular rings, both of which are essential for potential molecular qubits, have been transfered to the novel QD@Cr_7_Ni‐SH hybrids [15]. To confirm the spin‐groud state, low‐temperature X‐band (*ca*. 9.4 GHz) EPR spectroscopic studies were perform on these materials. We recall here that a low temperature is necessary because the *S* = 1/2 ground state of {Cr_7_Ni} MNMs is stabilized by intramolecular antiferromagnetic exchange involving the eight metals of the ring [40, 75], all of which are ochahedral and interconnected by fluoride and carboxylate ligands (Figure 1). Therefore, a 5 K temperature assures that this state is suficiently separated from the *S* = 3/2 excited state. As expected, the continuous‐wave (CW) EPR spectrum reveals a single resonance peak that is nicely simulated with the characteristic *g*‐values of *g*_x_ = *g*_y_ = 1.78 and *g*_z_ = 1.74 for magnetically isolated {Cr_7_Ni} rings (Figure 5a) [76]. This result provides compelling evidence that Cr_7_Ni‐SH rings are grafted onto the QD surface, as envisaged, without alteration to the antiferromagnetic core of the Cr_7_Ni molecules [40].

**FIGURE 5:**
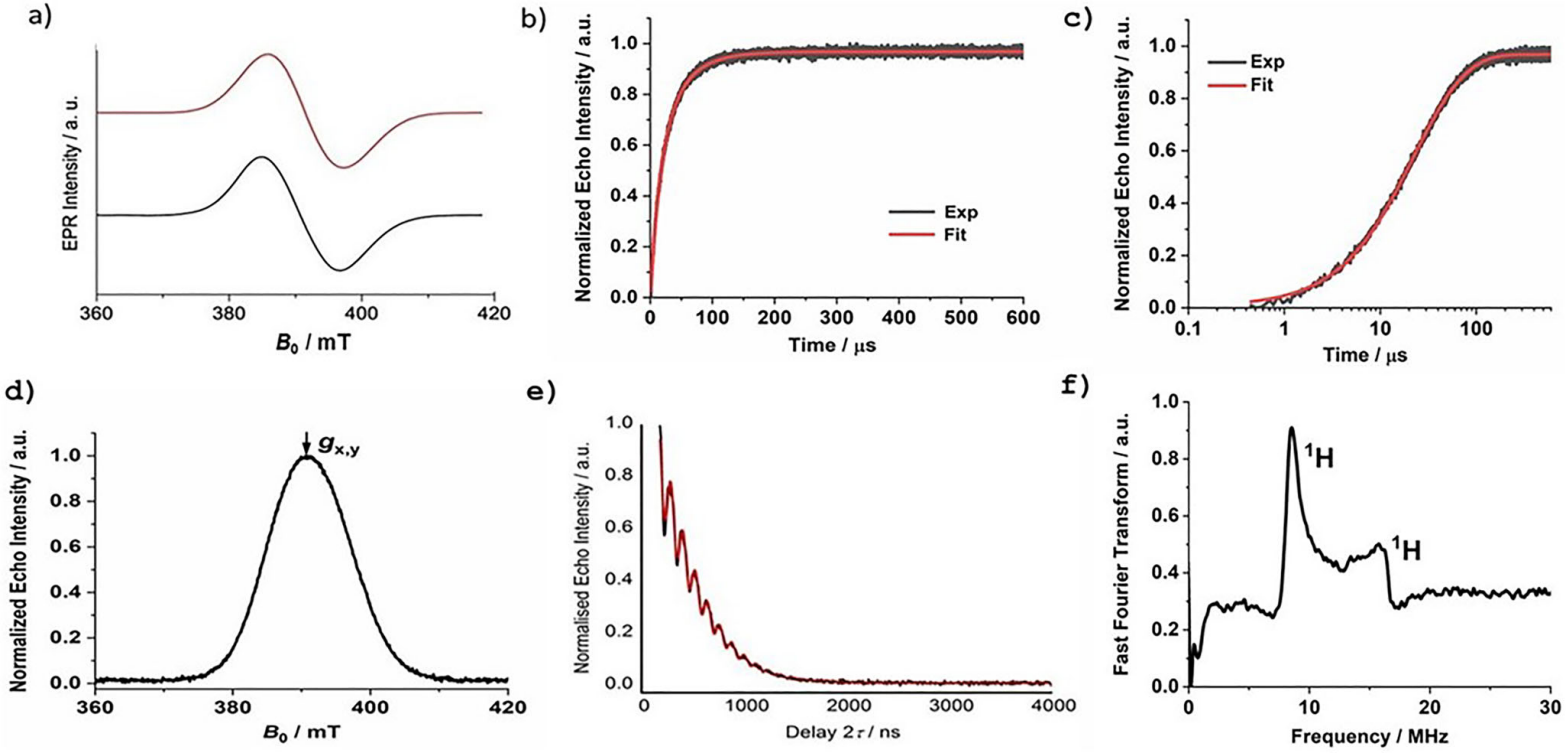
(a) Continuous‐wave X‐band EPR spectrum for QD@Cr_7_Ni‐SH in CHCl_3_ at 5 K (black trace) and its simulated version (red trace) with *S* = ½, *g*_x_ = g_y_ = 1.78 and *g*_z_ = 1.74. (b–f) Pulse EPR spectra for QD@Cr_7_Ni‐SH in CHCl_3_ at 2.6 K: (d) EDFS spectrum with the black arrow marking the field position corresponding to *g*_x,y_ at which further pulse measurements were conducted; (b) Inversion recovery curve (black trace), and its fit (red trace) to Equation (1), yielding spin lattice relaxation time constant, *T*_1_ = 41.3(5) µs, and spectral diffusion time *T*_SD_ = 15.8(3) µs; (c) Same inversion recovery data as (b) plotted in semilog fashion; (e) Two‐pulse electron spin echo decay measured with short pulses of π/2 = 16 ns and π = 32 ns, showing strong modulation from protons (ν_H_ = 16.6(1) MHz), and its simulation (red trace) using Equation (3); and (f) Fast Fourier Transform (FFT) of the ESEEM spectrum showing the characteristic peaks of ^1^H nuclear isotopes.

To explore the qubit‐potential of the MNMs attached to QDs, we performed pulse EPR measurements at X‐band (ca. 9.7 GHz). The echo‐detected field swept (EDFS) spectrum of QD@Cr_7_Ni‐SH (Figure 5d) resembles that recorded by CW EPR measurement (Figure 5a) except it is recorded as an absorption instead of derivative spectrum. This EDFS spectrum was measured with a primary Hahn‐echo pulse sequence with π/2 and π pulses of 16 ns and 32 ns length, respectively, and a pulse delay of 300 ns. During the measurement, the integral of the electron spin echo generated with the Hahn sequence was recorded as a function of the magnetic field. To determine the spin lattice relaxation (*T*_1_) and phase‐memory (*T*_m_) time constants, we performed three‐pulse magnetization inversion recovery measurements and two‐pulse echo decay measurements, respectively, at the field position corresponding to *g*_x,y_, that is at the top of the absorption peak generated in the EDFS measurement (Figure 5d). Figures 5b,c show the magnetization inversion recovery curve for QD@Cr_7_Ni‐SH at 2.6 K (black trace) and its fit to the bi‐exponential Equation (1) (red trace), yielding time constants of *T*_1, slow_ = 41.3(5) µs and *T*_1, fast_ = 15.8(3) µs (Table S2). The larger time constant, *T*_1, slow_, associates with spin lattice relaxation, and is typically termed *T*_1_ [2, 13]. The smaller time constant, *T*_1, fast_, is typically attributed to spectral diffusion (SD) in inversion recovery experiments [77, 78], thought that spin diffusion cannot be excluded [12, 21, 41]. For QD@Cr_7_Ni‐SH, SD relaxation is primarily facilitated by the multiple methyl groups of the pivalate ligands of the molecular {Cr_7_Ni‐SH} fragments [11, 21]. *T*_1_ is very long and in the limits observed for other molecular qubits, including {Cr_7_Ni} qubits [2, 12, 43]. Compared to the precursor ring **2** (*T*_1_ = 37.5(1) µs and *T*_SD_ = 14.8(1) µs at 2.46 K; Table S2), we record a small improvement in *T*_1_ which is to be expected considering the more rigid arrangement of the rings in QD@Cr_7_Ni‐SH (i.e., some of *^t^* Bu groups and/or pedant arm have their motion more restricted). However, these effects are minor as *T*_1_ for the single ring and QD@Cr_7_Ni‐SH is of the same order of magnitude and the relevant inversion recovery curves appear similar (Figure S2). The *T*_m_ time of QD@Cr_7_Ni‐SH was determined by fitting the timely decay of the Hahn echo (Figures 5e; Figures S4 and S5) generated by the standard π/2–τ–π–τ–echo pulse sequence, when the inter‐pulse delay τ was gradually incremented, to an exponential function described by Equations (2) and (3) [21, 41]. As expected, *T*_m_ increases with decreasing temperature allowing us to measure values as high as 629(4) ns in frozen CHCl_3_ solutions, which is comparable with that of the single ring **2** (673(1) at 2.46 K, Table S2), and within the range previously reported for {Cr_7_Ni} rings and their derivatives when these were suspended in similar ^1^H‐rich solvents [41, 43]. The fact that *T*_m_ remains almost unaffected after attaching the rings to the QD particle is a positive result indicating that one can form multi‐qubit arrays without the electron spin of one sub‐unit producing a catastrophic effect on another unit [79]. For short pulses (π/2 and π of 16 ns and 32 ns, respectively) we observed very strong electron spin echo envelope modulations (ESEEM) with some second harmonic content (Figures 5d and S3), which is indicative of coupling of the electron spin to nearby nuclear moments [80]. The nuclear frequency extracted from the modulation, 16.6(1) MHz, indicates that the relevant nuclei are protons [11], and thus, one can assume that hyperfine couplings to ^1^H nuclei contribute to decoherence. There are several sources of protons in QD@Cr_7_Ni‐SH, including the pivalate (Piv) ligands, the templating Pr_2_NH_2_^+^, and the phenyl groups of the tread. The rotational and vibrational motions of the forty‐five Me groups of Piv ligands in Cr_7_Ni‐SH are assumed to cause the most pronounced dephasing [41]. Nevertheless, this ESEEM effect can be suppressed by using longer, selective pulses of 64 ns π/2 length (Figure S4). The fact that the echo decay is well described by a stretched exponential function (Equation 2) with a stretching factor *s* of 1.18‐1.52 indicates that such nuclear modulation effects are only partially suppressed [41]. Above 8 K, the echo signal is lost rapidly due to thermal population of the *S* = 3/2 excited state of the antiferromagnetic {Cr_7_Ni‐SH} entities. It is noteworthy that the obtained *T*_1_ and *T*_2_ values for the hybrid molecule are comparable with those of colloidal Mn^2+^‐doped transition metal chalcogenide QDs explored by pulsed‐EPR [81]. Future studies will involve studying the MNMs while the QDs are photoirradiated.

## 3 Conclusion

In summary, a {Cr_7_Ni} ring derivative possessing a single pendant thiol group was successfully grafted onto the surface of CdSe/ZnS QDs via a ligand exchange method. The CdSe/ZnS QDs showed no significant change in optical properties post‐ligand exchange, displaying intense PL with a QY as high as 16.2%. Likewise the {Cr_7_Ni‐SH} rings attached to CdSe/ZnS QDs via ligand exchange have retained their characteristic magnetic behavior presenting robust coherence that is characteristic of their *S* = 1/2 electronic ground state stabilized by intra‐ring antiferromagnetic exchange, with a phase memory time of 0.6 µs. We anticipate that this memory time can be further improved through ligand and solvent deuteration and/or using more rigid versions of the heterometallic rings aimed to reduce decoherence from vibrational modes and nearby protons, as previously demonstrated by us [41]. Our current focus has been on probing the possibility of scaling molecular qubits using QDs as substrates which will open a suite of new possibilities for qubit optical manipulation and readout. We show that grafting of Cr_7_Ni MNMs to QDs provides a facile method for generating MNM assemblies without MNM agglomeration or alteration, raising the prospect of developing new materials with multimodal functionalities. It is envisaged that the development of colloidally stable MNM‐functionalized nanocrystals can provide a route to observing unique magneto‐optical interactions through tuning linker length to bring the Cr_7_Ni rings closer to the QD surface. Furthermore, ligand design will be explored in view of enhancing MNM coherence times and promoting MNM coupling at the nanoparticle surface. We are currently developing this approach toward incorporation of magnetically doped QDs, ideally with only one metal center like Mn^2+^ or Er^3+^ so that we take advantage of available pulse EPR methods (like DEER or RIDME spectroscopies) to interrogate the interactions between molecular ring qubits and QD hosts. This follows our recent studies of Mn‐doped QDs where we found that qubit lifetimes could be greatly expanded by growth of colloidal QDs from singly Mn‐doped molecular cluster seeds [4]. PL QYs and number of molecular qubits attached to QD can be further tuned by amending the size of QDs and the thickness of CdSe and ZnS monolayers.

## 4 Experimental Section

### 4.1 Materials and Methods

[*^n^* Pr_2_NH_2_][Cr_7_Ni(*μ*‐F)_8_(O_2_C*^t^* Bu)_16_], abbreviated as {Cr_7_Ni} (**1**), was prepared according to the literature procedure [39], but with [2NiCO_3_·3Ni(OH)_2_·4H_2_O] as the source of Ni(II) instead of the compound [Ni_2_(μ‐OH_2_) (O_2_C*^t^* Bu)_4_(HO_2_C*^t^* Bu)_4_] used in the original method, and with a reaction time of 24 h at 160°C. [*^n^* Pr_2_NH_2_][Cr_7_Ni(*μ*‐F)_8_(O_2_C*^t^* Bu)_15_(O_2_CC_12_H_8_OH)], abbreviated as {**Cr_7_Ni‐OH**} (**2**), was synthesized following a previously reported procedure [43].

#### 4.1.1 Instrumentation

High angle annular dark field (HAADF) scanning transmission electron microscopy (STEM) and energy dispersive x‐ray (EDX) spectrum imaging were performed using a probe side aberration corrected FEI Titan G2 80‐200 S/TEM “ChemiSTEM” instrument operated at both 200 kV and 80 kV with a convergence angle of 21 mrad. Samples were prepared by dropping a dilute nanoparticle solution onto a carbon coated copper grid. EDX spectra and spectrum images were acquired using the Titan's Super‐X SDD detector system and analyzed using the Bruker Esprit software. HAADF STEM images were acquired with a HAADF inner angle of ∼54 mrad using FEI TIA software and analyzed using Gatan Digital Micrograph and Image J software. EDX spectra quantification was performed in Bruker Esprit software using the Cliff‐Lorimer approach and with no absorption correction. Nanoparticle size distribution analysis was performed using Trainable Weka Segmentation software (see ESI for full experimental details).

PL emission spectra were collected using a GILDEN Photonics fluoroSENS spectrofluorometer at a 400 nm excitation wavelength, with a 2 nm resolution. QYs were measured using an integration sphere (Edinburgh Instruments). DLS measurements were performed at 25°C in chloroform using a Malvern ZetaSizer (Malvern Instruments Ltd, UK). Samples were diluted and passed through a 0.45 µm PTFE membrane filter (Millex) prior to measurements. Data was collected in automatic analysis mode, and the hydrodynamic diameter was calculated based on a number weighted percentage distribution after three runs.

Continuous‐wave (c.w.) electron‐paramagnetic resonance (EPR) measurements were performed at X‐band (*ca*. 9.5 GHz) on samples in chloroform at 5 K with a Bruker EMX 300 EPR spectrometer. Pulse EPR measurements at X‐band (ca. 9.7 GHz) were performed at 3 and 5 K on a Bruker ElexSys E580 spectrometer equipped with a Bruker split‐ring resonator and an Oxford CF 935 cryostat. Field corrections were applied for all data using Bruker Strong Pitch (g = 2.0028) as a reference. Experimental spectra were simulated using the EasySpin 6.0 software implemented in MATLAB [82]. Additional details on pulse EPR procedures and data analysis are presented in the Electron Paramagnetic Resonance Section.

#### 4.1.2 Syntheses

##### 4.1.2.1 CdSe QDs

The CdSe QD cores used in this study were synthesized following a previously reported protocol [65]. Once the growth period was complete, the heat source was removed. After reaching 60°C, hexane (30 mL) was injected into the reaction solution to prevent solidification. The QDs were isolated with the addition of acetone and centrifugation (10,000 rpm for 15 min). The supernatant was discarded and the pellet resuspended in chloroform. This washing/centrifugation procedure was repeated two more times. The final pellet was resuspended in chloroform and stored under N_2_ at room temperature. CdSe QD concentration and size was calculated based upon UV‐vis absorption spectra.

##### 4.1.2.2 CdSe/ZnS QDs

**CdSe/ZnS QDs** were synthesized using the SILAR method following a literature protocol [67]. The injection volume of each precursor was calculated based on equations described by Reiss et al. to shell a CdSe core with two monolayers of ZnS [83]. Octadecene (6 mL) and octadecylamine (2 g) were stirred at 100 °C under vacuum for 1 h. After being cooled to room temperature, CdSe QD stock solution (3 mL; 0.366 µmol) was injected and the reaction mixture was heated to 100 °C under vacuum, and then to 245 °C under N_2_. The precursors were alternately injected at 10 min intervals, and after the final injection, the temperature was raised to 260 °C and held for 30 min. Once complete, the reaction was allowed to cool. At 60 °C, hexane (20 mL) was injected into the reaction solution. The CdSe/ZnS QDs were precipitated using acetone, and isolated using centrifugation (10,000 rpm; 10 min). The pellet was then resuspended in chloroform, and this washing/centrifugation procedure was repeated two more times. The final pellet was suspended and stored in chloroform under N_2_.

##### 4.1.2.3 Cr_7_Ni‐SH (3)

A mixture of {Cr_7_Ni‐OH} (**2**) (1.20 g, 0.5 mmol), 4‐mercaptobenzoic acid (0.14 g, 0.5 mmol), dicyclohexylcarbodiimide (0.10 g, 0.5 mmol) and 4‐(dimethylamino)pyridine (0.06 g, 0.5 mmol) in THF (10 mL) was stirred for two days at room temperature under an argon atmosphere. The reaction mixture was filtered to remove the dicyclohexylurea formed and the solvent evaporated under reduced pressure. The resulting residue was purified by column chromatography. First toluene, followed by 60:1 toluene:ethyl acetate was used, which allowed {Cr_7_Ni‐SH} to be eluted, leaving the un‐reacted {Cr_7_Ni‐OH} at the top of the column. The solvent was removed under reduced pressure and a microcrystalline powder of {Cr_7_Ni‐SH} was obtained after recrystallization from Et_2_O/MeCN (0.70 g, 55 %). ESI‐MS (*m*/*z*): +2566 [M + Na]^+^; −2542 [M −H]^−^; 2441 [M‐*^n^* Pr_2_NH_2_]^–^. Elemental analysis (% calcd., % found for C_101_H_164_Cr_7_F_8_NNiO_34_S): C (47.70, 47.47), H (6.50, 6.48), Cr (14.31, 14.67), N (0.55, 0.50), Ni (2.31, 2.26).

##### 4.1.2.4 QD@Cr_7_Ni‐SH

For the ligand exchange, a 300‐fold excess of {Cr_7_Ni‐SH} (5.7 x10^−3 ^mmol) was stirred with CdSe/ZnS QDs (1.83 x10^−5 ^mmol) in 10 mL CHCl_3_ for 24 h under N_2._ Once complete, QD@Cr_7_Ni‐SH was aggregated with acetone (40 mL), and isolated using centrifugation (10,000 rpm; 30 mins). The pellet was resuspended in CHCl_3_, and the washing procedure repeated twice to remove unbound ligands. The final pellet was resuspended in CHCl_3_ (2 mL) and stored in under N_2._

#### 4.1.3 Electron Paramagnetic Resonance

Echo‐detected field‐swept (EDFS) spectra were recorded with a primary Hahn‐echo sequence (π/2‐τ‐π‐τ‐*echo)* [80], with π/2 and π microwave pulse lengths of 16 ns and 32 ns, respectively, fixed delay time τ = 300 ns, and varying the static *B*_0_ magnetic field. The maximum resonance intensity is observed at the field position associated to *g*_x,y_ = 1.78 (ca. 3910 G; Figure 5d).

Spin‐lattice relaxation time measurements were carried out using the magnetization inversion recovery pulse sequence (π‐*t*‐π/2‐τ‐π‐τ‐echo) [80], with π = 32 ns and τ = 320 ns and gradually increasing *t* (Figure 5b,c). The spin‐lattice relaxation time constant, *T*_1_, was determined by fitting the experimental data to Equation (1) [77]:

(1) $$Y\left(t\right)=Y\left(0\right)+Y_{1}e^{\left(-t/T_{1,\;\mathrm{slow}}\right)\;}+Y_{2}e^{\left(-t/T_{1,\mathrm{fast}}\right)}$$

where *Y*_1_ and *Y*_2_ are the amplitudes associated with a slower and a faster magnetization relaxation process, respectively, of which the slower process is typically attributed to the spin‐lattice relaxation, and the faster process to the occurrence of spectral diffusion (SD) [13, 19, 41].

Electron spin‐spin echo envelope modulation (ESEEM) measurements were carried out by gradually increasing the inter‐pulse delay τ of the primary Hahn echo sequence (π/2‐τ‐π‐τ‐*echo)* [80]. Strong ^1^H ESEEM is observed in the echo decays as a function of 2τ for π/2 and π pulses of 16 and 32 ns, respectively (Figures 5e and S2). Application of a Fast Fourier Transform (FFT) to the ESEEM data provided the frequency‐domain spectrum in Figure 5f, which is dominated by the spectral characteristics of ^1^H nuclei. ESEEM effects could be reduced by using more selective, longer microwave pulse lengths of *π/2* = 64 ns and *π* = 128 ns (Figure S3).

The phase memory time *T*_M_ was determined by least squares fitting of the echo decay with a stretched exponential using a solver based on the Levenberg‐Marquardt algorithm. The fitting function used was [41]:

(2) $$Y\left(2\tau \right)=Y\left(0\right)e^{\left(-2\tau /T_{m}\right)s}$$

or, for strongly modulated data,

(3) $$Y\left(2\tau \right)=Y\left(0\right)e^{\left(-2\tau /T_{m}\right)s}\left(1+\sin\left(\omega t+\phi \right)\right)$$

where *k* is the modulation depth, *ω* is the Larmor angular frequency of a nucleus *I* coupled to the electron spin, *ϕ* is the phase correction, *s* is the stretching parameter, *Y*(2*τ*) is the echo integral for a pulse separation *τ*, and *Y*(0) is the echo intensity extrapolated to *τ* = 0.

The calculated spin‐lattice relaxation time constant (*T_1_*), the spectral diffusion time constant (*T_SD_*), the phase memory time (*T_m_*), and the stretching parameter *(s)* are presented in Table S2.

## Conflicts of Interest

The authors declare no conflicts of interest.

## Supporting information



Additional data that support the findings of this study are available in the Supporting Information section of this article, which includes additional details on materials, instrumentation, and experimental protocols used in this study, additional EDX and EPR spectra, and quantum dot to ring ratio calculation methodology.
**Supporting File 1**: chem71619‐sup‐0001‐SuppMat.pdf.

## Data Availability

The data that support the findings of this study are available on request from the corresponding author. The data are not publicly available due to privacy or ethical restrictions.
