# Supplementary material for: Scaling Up Molecular Electron Spin Qubits Around a Core‐Shell Quantum Dot
Source: Chemistry. 2026 Aug 29;32(42):e71619. doi: 10.1002/chem.71619 (PMC13755140; doi:10.1002/chem.71619)
Supplement: Supplementary file 1 [file CHEM-32-e71619-s001.pdf]

Please do not adjust margins

Electronic Supplementary Information to

Scaling Up Molecular Electron Spin Qubits around a Core Shell Quantum Dot

Simon G. McAdams,[a] Jesus Ferrando-Soria,[a] Paul D. McNaughter,[a] Edward A. Lewis,[b] Sarah J. Haigh,[c] David J. Lewis,[a],[b] Richard E. P. Winpenny,*[a] Paul O'Brien, † [a],[b] and Floriana Tuna*[a],[d]

[a] Dr S. G. McAdams, Dr J. Ferrando-Soria, Dr P. D. McNaughter, Prof. R. E. P. Winpenny, Prof. P. O'Brien, Dr. F. Tuna
Department of Chemistry, University of Manchester, Oxford Road, M13 9PL, UK
E-mail: richard.winpenny@manchester.ac.uk; floriana.tuna@manchester.ac.uk

[b] Dr E. A. Lewis, Prof. D. J. Lewis, Prof. P. O'Brien
Department of Materials, University of Manchester, Oxford Road, M13 9PL, UK

[c] Prof. S. J. Haigh
National Graphene Institute, University of Manchester, Oxford Road, M13 9PL, UK

[d] Dr F. Tuna
Photon Science Institute, University of Manchester, Oxford Road, M13 9PL, UK

† Died 16 October 2018

Contents

Supplementary information to 1
1. Materials 2
2. Instrumentation 2
3. Synthesis 3
4. Additional EDX data 4
5. Quantum Dot to Ring Ratio Calculations 5
6. Electron Paramagnetic Resonance 6
6. References 8

Please do not adjust margins

Please do not adjust margins

1. Materials

All materials and chemicals were purchased from Sigma-Aldrich, except zinc oxide (BDH Reagents); and octadecylamine (ACROS organics). All chemicals were used as received. $[^{n}Pr_2NH_2][Cr_7Ni(\mu\text{-}F)_8(O_2C^{t}Bu)_{16}]$ was prepared according to literature procedure [1], but with $[2NiCO_3\cdot 3Ni(OH)_2\cdot 4H_2O]$ as the source of Ni(II) instead of the compound $[Ni_2(\mu\text{-}OH_2)(O_2C^{t}Bu)_4(HO_2C^{t}Bu)_4]$ used in the original method, and with a reaction time of 24 h at 160 °C. $[^{n}Pr_2NH_2][Cr_7Ni(\mu\text{-}F)_8(O_2C^{t}Bu)_{15}(O_2CC_{12}H_8OH)]$, {**$Cr_7$Ni-OH**}, was synthesised following a literature procedure [2]. Dry tetrahydrofuran was obtained by passing the solvent (HPLC grade) through an activated alumina column on a PureSolvTM solvent purification system (InnovativeTechnologies Inc., MA). Column chromatography was carried out using Silica 60 A (particle size 35-70 *μ*m, Fisher, UK) as the stationary phase, and TLC was performed on pre-coated silica gel plates (0.25 mm thick, 60 F_{254}, Merck, Germany).

2. Instrumentation

High resolution TEM (HRTEM) imaging, high angle annular dark field (HAADF) scanning transmission electron microscopy (STEM) imaging, and energy dispersive X-ray (EDX) spectrum imaging were performed using a probe side aberration corrected FEI Titan G2 80-200 S/TEM "ChemiSTEM™" instrument operated at 200 kV with a probe current of ~800 pA and a convergence angle of 21 mrad. Samples were prepared by dropping dilute nanoparticle solution onto a carbon coated copper grid. EDX spectra and spectrum images were acquired using the Titans Super-X SDD detector system (total collection angle ~0.7srad) and analysed using the Bruker Esprit software. HAADF STEM images were acquired with a HAADF inner angle of ~54 mrad using FEI TIA software and analysed using Gatan Digital Micrograph and Image J software. EDX spectra quantification was performed in Bruker Esprit software using the Cliff-Lorimer approach and with no absorption correction. Nanoparticle size distribution analysis was performed using Trainable Weka Segmentation software [3] that is part of the open-source Image J / Fiji package [4].

Elemental analysis measurements were performed by the University of Manchester Micro Analytical Laboratory. ICP-MS measurements were performed by the University of Manchester School of Earth, Atmospheric and Environmental Sciences.

UV–VIS absorption spectra were collected using a double beam UV-1800 Shimadzu UV-vis spectrophotometer between 200 – 900 nm. Samples were measured in quartz cuvettes with path length of 1 cm, and pure solvent was used as a blank. PL emission spectra were collected using a GILDEN Photonics fluoroSENS spectrofluorometer at a 400 nm excitation wavelength, with a 2 nm resolution. Quantum yields were measured using an integration sphere (Edinburgh Instruments).

Dynamic light scattering (DLS) measurements were performed at 25 °C in chloroform using a Malvern ZetaSizer (Malvern Instruments Ltd, UK). Samples were diluted and passed through a 0.45 µm PTFE membrane filter (Millex®) prior to measurements. Data was collected in automatic analysis mode; and the hydrodynamic diameter calculated based on a number weighted percentage distribution after three runs.

This journal is © The Royal Society of Chemistry 20xx

Please do not adjust margins

Please do not adjust margins

Continuous-wave (c.w.) electron-paramagnetic resonance (EPR) measurements were performed at X-band (*ca*. 9.5 GHz) on samples in $CHCl_3$ at 5 K with a Bruker EMX 300 EPR spectrometer. Pulse EPR measurements at X-band (ca. 9.7 GHz) were performed at 3-5 K on a Bruker ElexSys E580 spectrometer equipped with a Bruker split-ring resonator and an Oxford CF 935 cryostat. The Q value of the over-coupled resonator was *ca*. 100. Two pulse spin echo decays were measured using the pulse sequence (π/2-τ-π-τ-echo) [5], with pulse lengths of 16 /32 ns or 64 /128 ns and a gradually incremented inter-pulse delay τ. The T_1 spin-lattice relaxation time was measured with the inversion recovery pulse sequence (π-t-π/2-τ-π-τ-echo) [5], with variable t and fixed τ (see section 6 for more details).

3. Synthesis

3.1 CdSe quantum dot synthesis

The 3.4 nm CdSe QD cores were synthesised following a previously reported protocol [6]. Once the growth period was complete, the heat source was removed and the reaction cooled. When the temperature reached 60°C, hexane (30 mL) was injected into the reaction solution to prevent solidification. The QDs were isolated with the addition of acetone and centrifugation (10,000 rpm for 15 minutes). The supernatant was discarded and the pellet resuspended in chloroform. This washing/centrifugation procedure was repeated two more times. The final pellet was resuspended in chloroform and stored under N_2 at room temperature. CdSe QD concentration and size was calculated based upon UV-vis absorption spectra [7].

3.2 CdSe/ZnS quantum dot synthesis

CdSe/ZnS QDs were synthesised using the SILAR method following a previous method protocol [8]. The injection volume of each precursor was calculated based on equations described by Reiss *et al.* to shell a CdSe core with two monolayers of ZnS [9]. To synthesise CdSe/ZnS QDs, octadecene (6 mL) and octadecylamine (2 g) were stirred in a 100 ml three-necked flask, and heated to 100 °C under vacuum, and held for 1 hour before cooling to room temperature. Next, the CdSe QD stock solution (3 mL; 0.366 μmol) was injected into the reaction flask using standard air-free techniques. The reaction was heated to 100 °C under vacuum and held for 30 minutes, and then heated to 245 °C under N_2. The precursors were alternately injected at 10 minute intervals, and after the final injection, the temperature was raised to 260 °C and held for 30 minutes. Once complete, the reaction was allowed to cool. When the temperature reached 60 °C, hexane (20 mL) was injected into the reaction solution. The CdSe/ZnS QDs were precipitated using acetone, and isolated using centrifugation (10 000 rpm; 10 minutes). The pellet was then resuspended in chloroform, and this washing/centrifugation procedure was repeated two more times. The final pellet was suspend in chloroform and stored under N_2. In addition to the expected increase in diameter from 3.3 nm to 4.2 ± 0.37 nm (one ZnS monolayer is equivalent to a theoretical increase in radius of 0.31 nm) and associated red-shift in PL, the quantum yield increased from 5.9% (CdSe) to 15.1% (CdSe/ZnS) after shelling CdSe with two ZnS monolayers. The presence of Zn and S was confirmed using HAADF imaging and EDX spectrum imaging on the QD@Cr_7Ni-SH sample.

This journal is © The Royal Society of Chemistry 20xx

Please do not adjust margins

3.3 QD@Cr_7Ni-SH synthesis

For the ligand exchange, a 300 fold excess of {**Cr_7Ni-SH**} (5.7 x10^{-3} mmol) was stirred with CdSe/ZnS QDs (1.83 x10^{-5} mmol) in 10 ml $CHCl_3$ for 24 hours under N_2. Once complete, **QD@Cr_7Ni-SH** was aggregated with acetone (40 ml), and isolated using centrifugation (10 000 rpm; 30 mins). The pellet was resuspended in $CHCl_3$, and the washing procedure repeated twice to remove unbound ligands. The final pellet was resuspended in $CHCl_3$ (2 ml) and stored in under N_2.

4. Additional EDX Data for QD and QD@Cr_7Ni-SH

The stability of the functionalised QDs limits the counts achievable in the EDX spectrum images. Typical data was acquired using a 200pA probe current, 50 us dwell time and a total acquisition time of 5 minutes. The HAADF image was monitored continuously during acquisition and imaging ceased as soon as changes were observed. The sum spectrum used for quantification contained >370, 000 counts. Quantification was performed to determine the ratio of Cd:Zn:Cr (Table S1), whilst deconvolving Se and S (from the QDs) and Cu, C, O and Si (caused by the presence of the TEM support grid). However we note that the Cr containing rings are likely to be less robust to prolonged electron beam exposure than the CdSe-ZnS QDs and preferential sputtering of the rings may be responsible for the slight reduction in Cr content we observe by EDX spectrum quantification than ICP-OES.

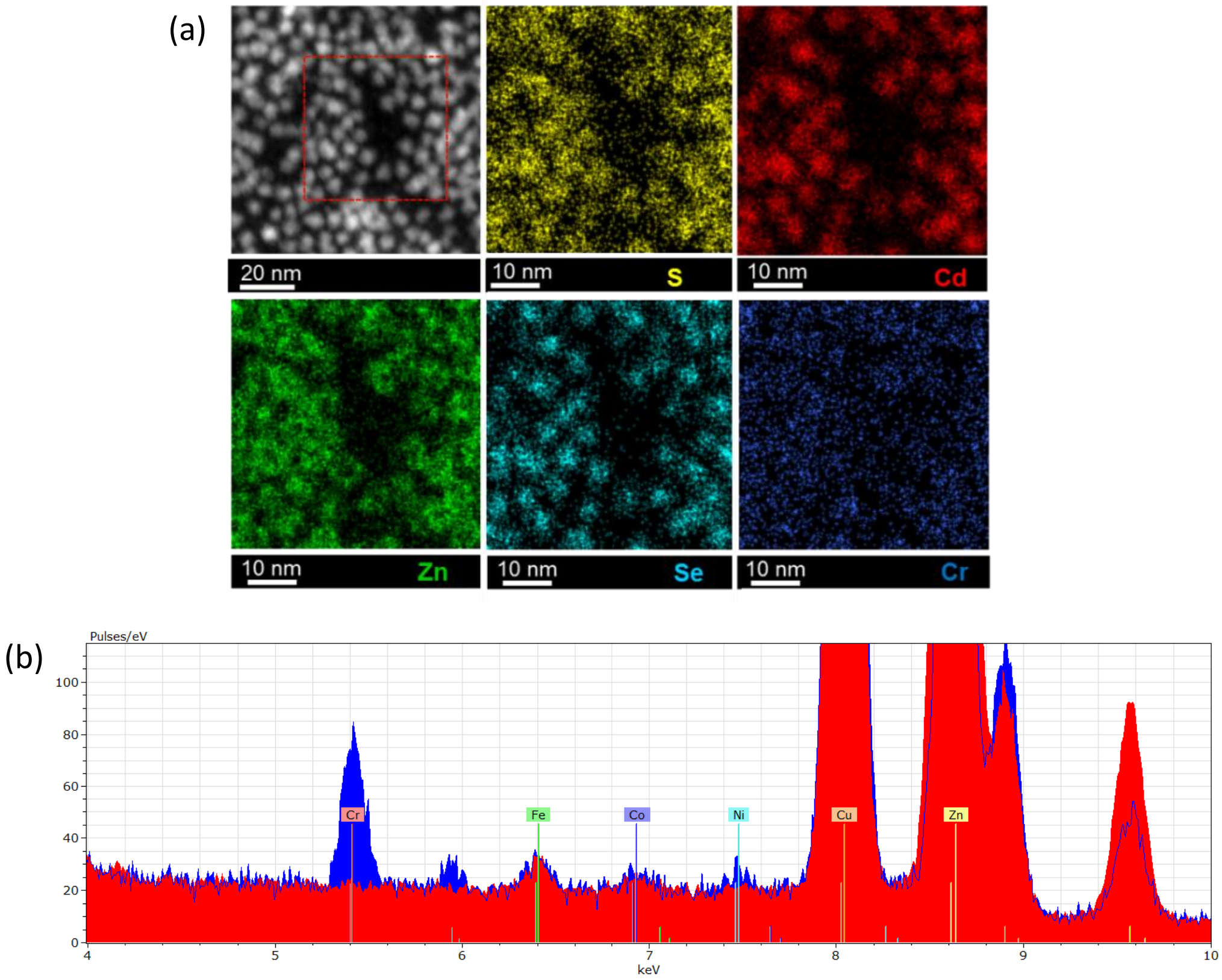

Figure S1. (a) Individual EDX of QD@Cr_7Ni-SH; and (b) Comparison of spectrum shown in Figure 2 (blue) and the spectrum of another comparable quantum dot sample (red) analysed using the same TEM grid and microscope. The small Fe and Co peaks arise from the pole piece of the microscope, Cu counts arise from grid bars, and Zn from the quantum dots. It is clear that Cr seen in our spectra arises from the functionalisation of the quantum dots and is not intrinsic to either the microscope or TEM grid.

This journal is © The Royal Society of Chemistry 20xx

Please do not adjust margins

ICP-OES and EDX quantification values for Cr, Zn, and Cd from QD@Cr_7Ni-SH are reported in Table S1

Table S1. EDX quantification and ICP-OES of QD@Cr_7Ni-SH

Element	*EDX (%)*	*ICP-OES (%)*
Cd	42.3 ±1.5	46.9
Zn	52.7 ±1.5	43.2
Cr	5 ± 0.5	9.9

5. Quantum Dot to Ring Ratio Calculations

ICP-OES gives absolute masses of Cd and Cr in a QD@Cr_7Ni-SH sample as 1.13×10^{-5} grams and 1.1×10^{-6} grams, respectively. There are 354 Cd atoms per 3.3 nm CdSe core as calculated using the following equation [10]:

$N_{atoms} = (\pi\ \rho\ r^3 / 6M) * N_A$

Where N_{atoms} is the number of CdSe units; ρ the density ($g.nm^{-3}$) r the radius (nm); M the molecular weight ($g.mol^{-1}$) and N_A Avagadro's number.

Form this it was possible to calculate the number of quantum dots in the sample and, considering there are 7 Cr atoms per ring, the ratio of QDs to rings as 1 to 10.64, or 1 to ~11.

To approximate the number of {**Cr_7Ni-SH**} rings that can bind to the surface of 4.2 nm CdSe/ZnS QDs, {Cr_7Ni-SH} was modelled as a 'ligand cone' [11]. The diameter of the {**Cr_7Ni**} ring is ~ 1.8 nm [12]; and the linker length estimated as ~1.4 nm based on DLS data. Thus, the ligand cone has a base a radius of 1.8 nm, and a height of 1.4 nm. The maximum number of ligand cones that can fit on the surface of a spherical particle is estimated by dividing the area of a sphere, with a radius equal to the QD (2.1 nm) plus the linker length (1.4 nm), by the cone-base area (10.18 nm^2) [11]. This gives an estimation of ~15 {**Cr_7Ni-SH**} rings per QD.

This journal is © The Royal Society of Chemistry 20xx

Please do not adjust margins

6. Electron Paramagnetic Resonance

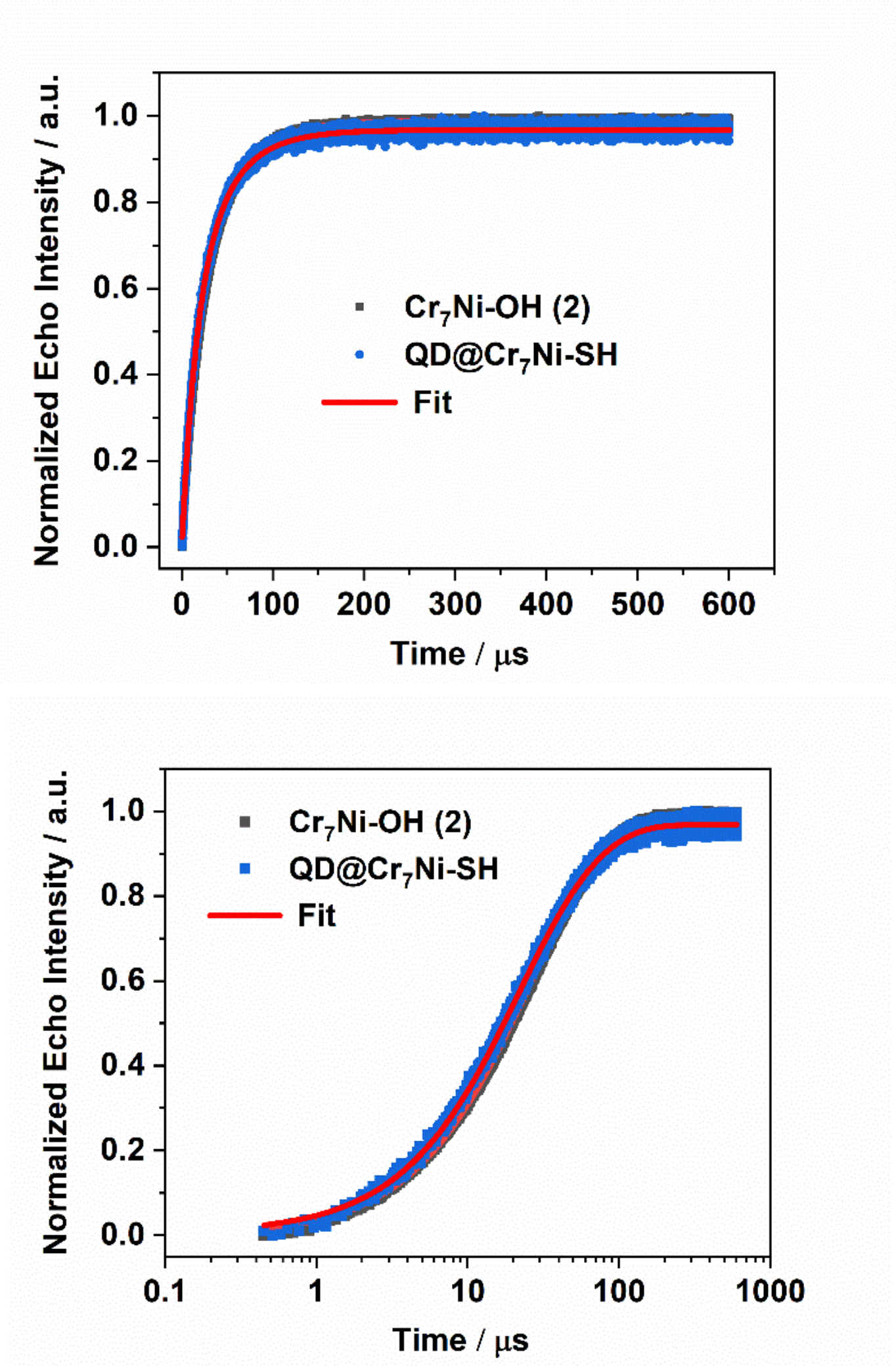

Figure S2. Comparison of the inversion recovery curves for **2** and **QD@Cr$_7$Ni-SH** in chloroform, measured at 2.5 K at X-band (ca 9.7 GHz) at the maximum resonance field for the ring, using the inversion recovery sequence with pulses of $\pi/2$ = 16 ns and π = 32 ns, and pulse delay of τ = 240 ns. The red trace is the fit to a bi-exponential function (Eq.1 in the main text), yielding the parameters in Table S2.

 This journal is © The Royal Society of Chemistry 20xx

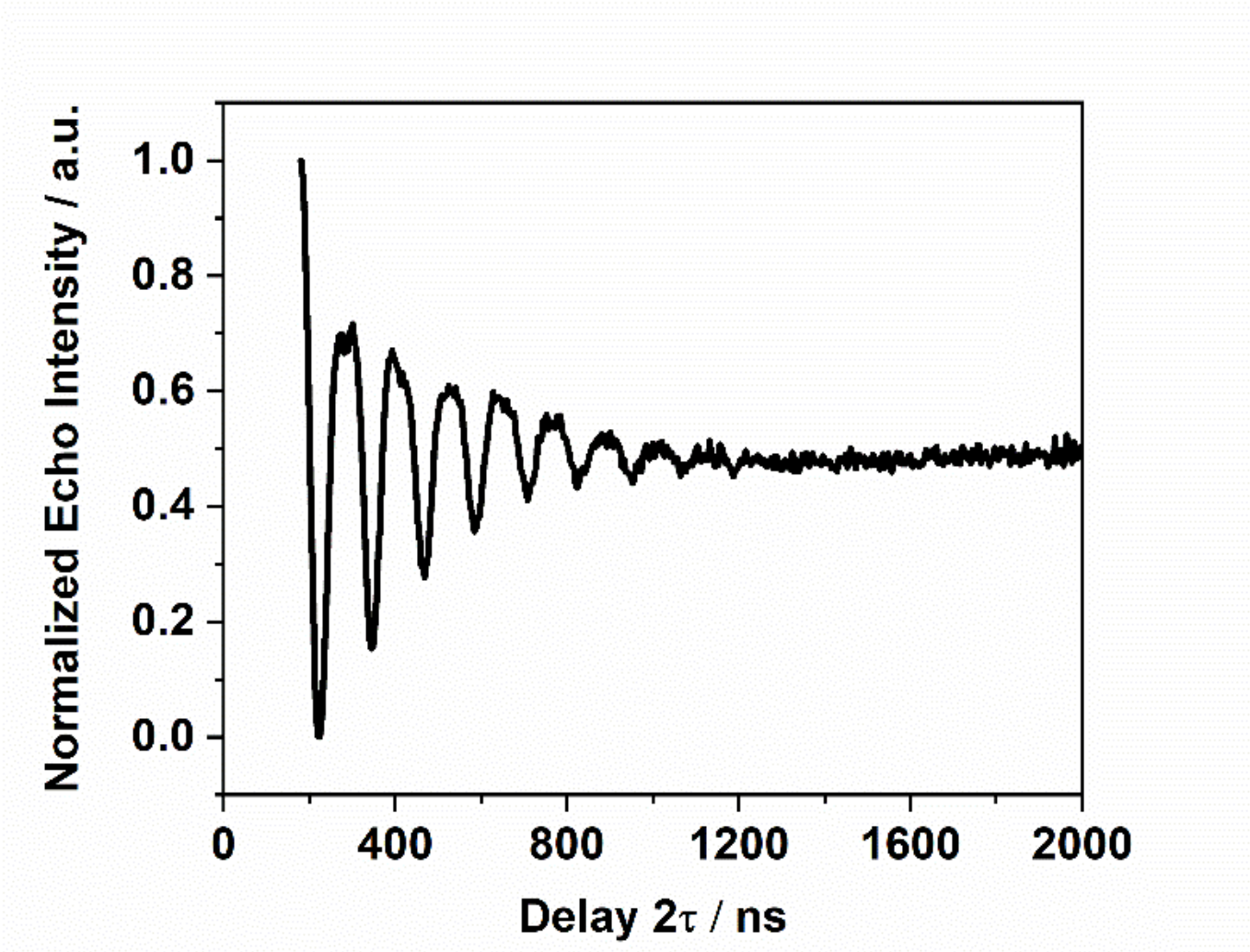

Figure S3. Two pulse electron spin-echo envelope modulation (ESEEM) spectrum at X-band for **QD@Cr_7Ni-SH** in diluted chloroform solution at the maximum resonance field for the ring, recorded with pulses of $\pi/2$ = 16 ns and π = 64 ns, and pulse delay of τ = 180 ns.

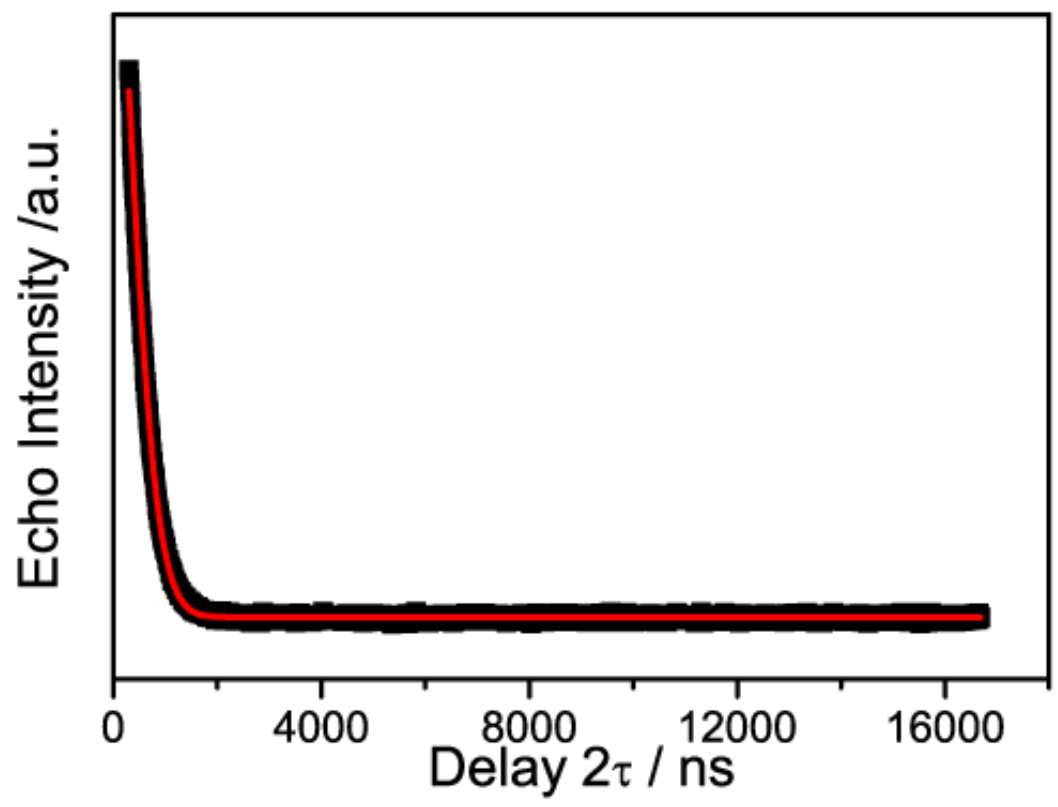

Figure S4. Two pulse electron spin-echo decays at X-band for **QD@Cr_7Ni-SH** in diluted chloroform solutions at the maximum resonance field for the ring, recorded with pulses of π = 128 ns. The red lines represent fits to Eq. 2, $Y(2\tau) = Y(0)e^{(-2\tau/T_m)s}$, with T_m and s values listed in Table S2.

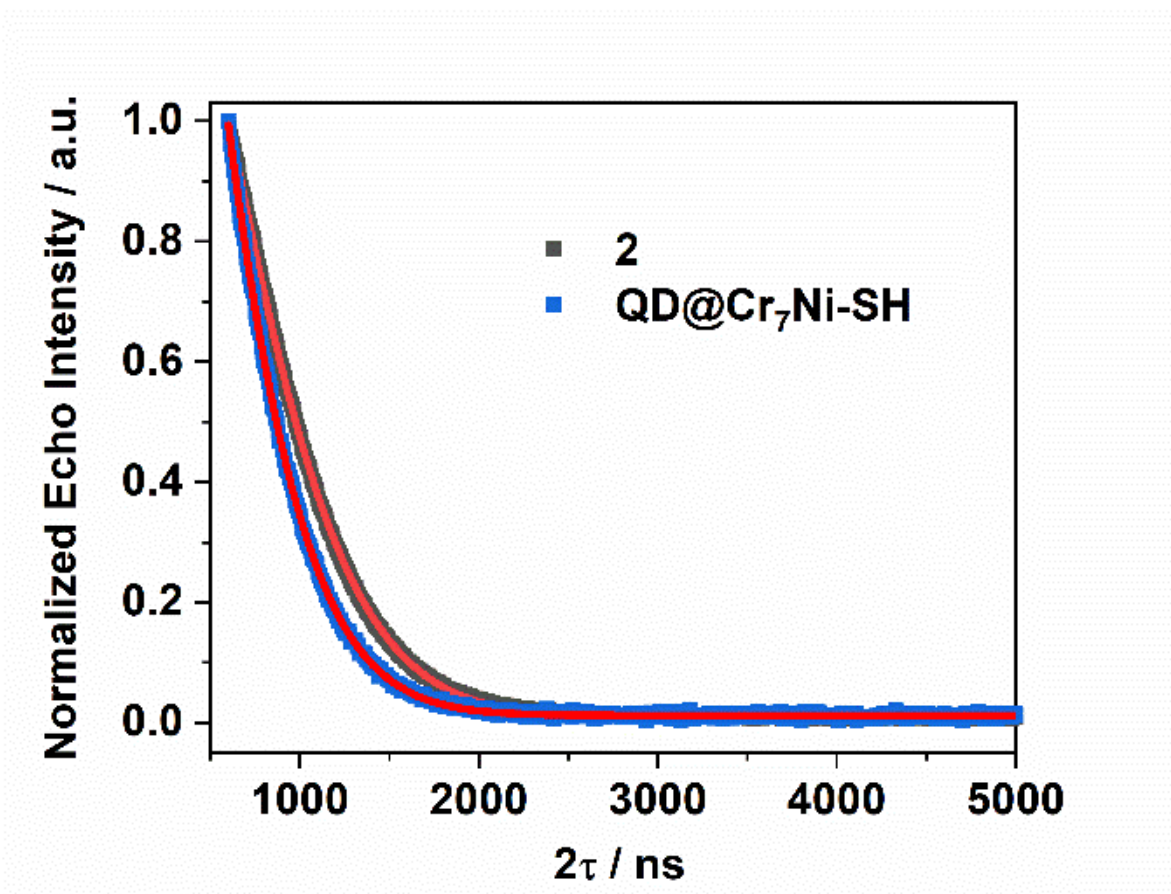

Please do not adjust margins

Figure S5. Comparison of the two pulse electron spin-echo decays at X-band for molecular ring **2** and **QD@Cr$_7$Ni-SH** in chloroform at the maximum resonance field for the ring, recorded with pulses of $\pi/2 = 64$ *ns and* $\pi = 128$ ns, and a pulse delay of 300 ns. The red lines represent fits to Eq. 2, $Y(2\tau) = Y(0)e^{(-2\tau/T_m)s}$, with T_m and *s* values listed in Table S2.

Table S2. X-band pulsed-EPR data at 3 K for **QD@Cr$_7$Ni-SH** in $CHCl_3$

Compound	T / K	$T_{1,\text{ slow}}$ / μs	$T_{1,\text{ fast}}$ / μs	T_m / ns	*s*
QD@Cr$_7$Ni-SH	2.6<br>3.0<br>5.5	41.3 ± 0.5	15.8 ± 0.3	629 ± 4<br>586 ± 1<br>305 ± 9	1.52<br>1.34<br>1.18
Cr$_7$Ni-OH (**2**)	2.46	37.5 ± 0.1	14.8 ± 0.1	673 ± 1	1.48

The time constants $T_{1,\text{slow}}$ and $T_{1,\text{fast}}$ in Table S2 were determined by fitting the inversion recovery data in Figure 5 b to a biexponential Eq. 1 (main text). T_m and s are obtained from the fitting of the data presented in Figures S4 and S5 to the stretch function Eq. 2 (main text).

7. References

1 F. K. Larsen, E. J. L. McInnes, H. E. Mkami, J. Overgaard, S. Piligkos, G. Rajaraman, E. Rentschler, A. A. Smith, G. M. Smith, V. Boote, M. Jennings, G. A. Timco and R. E. P. Winpenny, *Angew. Chem. Int. Ed.*, 2003, **42**, 101–105.

2 J. Ferrando-Soria, A. Fernandez, E. Moreno Pineda, S. A. Varey, R. W. Adams, I. J. Vitorica-Yrezabal, F. Tuna, G. A. Timco, C. A. Muryn and R. E. P. Winpenny, *J. Am. Chem. Soc.*, 2015, **137**, 7644–7647.

3 M. Hall, E. Frank, G. Holmes, B. Pfahringer, P. Reutemann and I. H. Witten, *SIGKDD Explor Newsl*, 2009, **11**, 10–18.

4 J. Schindelin, I. Arganda-Carreras, E. Frise, V. Kaynig, M. Longair, T. Pietzsch, S. Preibisch, C. Rueden, S. Saalfeld, B. Schmid, J.-Y. Tinevez, D. J. White, V. Hartenstein, K. Eliceiri, P. Tomancak and A. Cardona, *Nat. Methods*, 2012, **9**, 676–682.

5 A. Schweiger and G. Jeschke, "*Principles of Pulse Electron Paramagnetic Resonance*“, Oxford University Press, New York, 2001.

6 Q. Sun, Y. A. Wang, L. S. Li, D. Wang, T. Zhu, J. Xu, C. Yang and Y. Li, *Nat. Photonics*, 2007, **1**, 717–722.

7 W. W. Yu, L. Qu, W. Guo and X. Peng, *Chem. Mater.*, 2003, **15**, 2854–2860.

8 R. Xie, U. Kolb, J. Li, T. Basché and A. Mews, *J. Am. Chem. Soc.*, 2005, **127**, 7480–7488.

9 P. Reiss, M. Protière and L. Li, *Small*, 2009, **5**, 154–168.

10 X. Liu, M. Atwater, J. Wang and Q. Huo, *Colloids Surf. B Biointerfaces*, 2007, **58**, 3–7.

11 C. Bullen and P. Mulvaney, *Langmuir*, 2006, **22**, 3007–3013.

12 V. Corradini, R. Biagi, U. del Pennino, V. De Renzi, A. Gambardella, M. Affronte, C. A. Muryn, G. A. Timco and R. E. P. Winpenny, *Inorg. Chem.*, 2007, **46**, 4937–4943.

This journal is © The Royal Society of Chemistry 20xx

Please do not adjust margins
